# Susceptibility Pattern and Distribution of Oxacillinases and* bla*_PER-1_ Genes among Multidrug Resistant *Acinetobacter baumannii* in a Teaching Hospital in Iran

**DOI:** 10.1155/2015/957259

**Published:** 2015-12-31

**Authors:** Sareh Bagheri Josheghani, Rezvan Moniri, Farzaneh Firoozeh, Mojtaba Sehat, Yasaman Dasteh Goli

**Affiliations:** ^1^Department of Microbiology and Immunology, Faculty of Medicine, Kashan University of Medical Sciences, Kashan 87159 85191, Iran; ^2^Anatomical Sciences Research Center, Kashan University of Medical Sciences, Kashan 87159 85191, Iran; ^3^Trauma Research Center, Kashan University of Medical Sciences, Kashan 87159 85191, Iran; ^4^University of Maryland, College Park, MD 20742, USA

## Abstract

*Acinetobacter baumannii (A. baumannii)* is an important nosocomial pathogen in healthcare institutions. *β*-Lactamase-mediated resistance is the most common mechanism for carbapenem resistance in* A. baumannii*. The aim of this study was to determine the antibiotic resistance pattern, to detect* OXA* encoding genes, class A, *bla*_PER-1_, and to detect the presence of ISA*ba*1. A total of 124* A. baumannii* isolates were collected from hospitalized patients in a teaching hospital in Kashan, Iran. The susceptibility of isolates to different antibiotics was determined by disk-diffusion method. PCR was used to detect *bla*_PER-1_, *bla*_OXA-23_, *bla*_OXA-24_, *bla*_OXA-51_, *bla*_OXA-58_, and ISA*ba*1 genes. All isolates were resistant to ceftazidime, ceftriaxone, and cefotaxime. All of the isolates revealed susceptibility to polymyxin B and colistin. Ninety-six percent of the isolates were extensive drug resistance (XDR), 5.6% extended spectrum beta-lactamase (ESBL), and 54.8% metallo-beta-lactamase (MBL). All isolates were positive for *bla*_OXA-51_ and ISA*ba*1. *bla*_OXA-23_,  *bla*_OXA-24_, and *bla*_OXA-58_ were found in 79.8%, 25%, and 3.2%, respectively. The frequency rate of *bla*_PER-1_ gene was 52.4%. Multidrug resistant* A. baumannii* isolates are increasing in our setting and extensively limit therapeutic options. The high rate presence of class D carbapenemase-encoding genes, mainly *bla*_OXA-23_ carbapenemases, is worrying and alarming as an emerging threat in our hospital.

## 1. Introduction

Multidrug resistant* A. baumannii* is a main cause of hospital acquired infections and recognized to cause a wide spectrum of life-threatening diseases. This organism is resistant to almost all frequently accessible antibiotics which limits treatment options [1]. Emergence of resistance during medication for* A. baumannii* infections may occur and yields increasing rates of morbidity, mortality, and therapeutic costs [2].* A. baumannii* is capable of accumulating multiple antibiotic resistance genes, leading to development of multidrug resistant or extensively drug resistant strains. Acquired resistance characteristics occur as consequences from mutation or acquisition of exogenous resistance determinants and can be mediated by different mechanisms, including beta-lactamases, alterations in cell-wall channels, and efflux pumps. The most worrying clinical resistance mechanism has been acquisition of serine and metallo-beta-lactamases, which present resistance to carbapenems [3]. Therefore, this study was performed to identify the prevalence of beta-lactamases producing multidrug resistant* A. baumannii* and the molecular characterization of prevalent genes among them, with the purpose of improving the therapeutic options and decreasing the morbidity and mortality in Beheshti Hospital in Kashan, Iran.

## 2. Materials and Methods

### 2.1. Sample Collection

From June 2013 to November 2014, a descriptive study was conducted with various clinical samples collected from a tertiary care hospital in Kashan, Iran. A total of 124* A. baumannii* isolates were collected from clinical samples including tracheal tube 63 (50.8%), blood, 28 (22.6%), sputum, 10 (8.1%), pleural fluid, 7 (5.6%), urine, 7 (5.6%), cerebrospinal fluids, 4 (3.2%), urine catheter, 3 (2.4%), and wounds, 2 (1.6%), from hospitalized patients. The Ethics Committee of Kashan University of Medical Sciences approved the study protocol.

*Bacterial Isolates*.* A. baumannii* isolates were identified by using MICROGEN GNA+B (Microgen Bioproducts Co., UK).

### 2.2. Determination of Antibiotic Resistance Patterns of* A. baumannii*

Antibiotic sensitivity test of the isolates was determined using the Kirby-Bauer disk-diffusion breakpoint assay on Mueller-Hinton agar (Merck, Germany); and the cultures were incubated for 24 h at 37°C. Bacteria were classified as susceptible, intermediate, or resistant to antibiotics in accordance with the current Clinical Laboratory Standard Institute recommendations [4]. Piperacillin (100 *μ*g), ampicillin-sulbactam (10/10 *μ*g), piperacillin-tazobactam (100/10 *μ*g), cefotaxime (30 *μ*g), ceftazidime (30 *μ*g), ceftriaxone (30 *μ*g), cefepime (30 *μ*g), imipenem (10 *μ*g), meropenem (10 *μ*g), amikacin (30 *μ*g), gentamicin (10 *μ*g), ciprofloxacin (5 *μ*g), levofloxacin (5 *μ*g), tetracycline (30 *μ*g), trimethoprim-sulfamethoxazole (1.25/23.75 mg), colistin (10 *μ*g), and polymyxin B (300 IU) disks were purchased from MAST, Merseyside, UK.* Escherichia coli* ATCC 25922 were used as the quality control strain in every susceptibility test.

*MDR, XDR, and PDR Definitions*. Multidrug resistant (MDR) was applied when the isolate is nonsusceptible to at least one antibacterial agent in ≥3 of the following classes of antibiotics including quinolones, broad-spectrum cephalosporins, beta-lactamaseinhibitor/beta-lactams, aminoglycosides, tetracyclines, trimethoprim-sulfamethoxazole, and carbapenems. Extensive drug resistance (XDR) is defined as bacterial isolates remaining susceptible to only one or two categories. Pan-drug resistant (PDR) is defined as bacterial isolates with nonsusceptibility to all agents in all of the antimicrobial categories (i.e., no agents tested as susceptible for the organism). Thus, a bacterial isolate that is characterized as XDR will also be categorized as MDR. Similarly, a bacterial isolate would have to be XDR in order for it to be further defined as PDR [5].

### 2.3. Phenotypic Tests for Detection of ESBLs

ESBL was detected by double-disk-diffusion test using cefotaxime (30 *μ*g) and with cefotaxime/clavulanic acid (CTX 30 *μ*g + CA 10 *μ*g per disk) (MAST, Merseyside, UK). In accordance with the Clinical and Laboratory Standards Institute (CLSI) recommended guidelines, an increase in zone diameter ≥5 mm in the presence of clavulanic acid indicated presence of the extended spectrum-*β*-lactamases (ESBLs) in test organism [6].

### 2.4. Phenotypic Tests for the Detection of MBL

Imipenem-resistant isolates were screened for producing metallo-beta-lactamase (MBL). The double-disk synergy test (DDST) was performed for identification of MBL by imipenem (10 *μ*g), alone and in combination with EDTA (750 *μ*g/disk) (ROSCO, Denmark). An increase in the zone diameter of ≥7 mm around imipenem plus EDTA disk compared to that of imipenem disk alone was considered as positive for MBL production [6].

### 2.5. PCR Amplification of the Beta-Lactamase-Encoding Genes

A series of PCR reactions were performed to detect the different Ambler class* bla* genes. Primers were designed to amplify the following carbapenemase-encoding genes using a multiplex PCR: class D: *bla*_OXA-23_, *bla*_OXA-24_, *bla*_OXA-51_, and *bla*_OXA-58_; class A: *bla*_PER-1_ [7, 9]. IS elements (ISA*ba*1) were amplified by PCR using previously described methods [8]. All isolates were subjected to the multiplex PCR to detect *bla*_OXA-23_, *bla*_OXA-24_, *bla*_OXA-51_, and *bla*_OXA-58_ genes. The primers used for the analysis are listed in Table 1.

PCR products run on 1.0% agarose gel, stained with ethidium bromide, and photographed by UV illumination (Ingenius, Syngene). The size of the PCR product was compared with 100 bp DNA ladder (Bioneer, Korea).* A. baumannii* ATCC 19606 was used as reference strain.

*Sequencing Method*. Sequencing was done by Sanger's method (Applied Biosystems 3730/3730xl DNA Analyzers Sequencing; Bioneer). The sequences were analyzed using ChromasPro version 1.7.5 Technelysium (http://www.technelysium.com.au/). GenBank accession numbers for *bla*_OXA-23_, *bla*_OXA-24_, *bla*_OXA-51_, and *bla*_PER-1_ are KP462887, KP462888, KP462889, and KP462892.

### 2.6. Statistical Analysis

Statistical data analysis was conducted using SPSS software version 19 (SPSS Inc., Chicago, IL). The chi-square test was used to compare antibiotic resistance rates in this study. *P* < 0.05 was considered statistically significant.

## 3. Results

The mean age of the studied patients was 54.2 ± 18.1 years, which ranged from 23 to 95 years. The majority of patients (61.3%) were male, 76 versus 48 female. The number and rates of isolates from different wards of the hospital were as follows: ICU, 69 (55.6%); Internal Medicine, 26 (21%); Emergency Room, 23 (18.5%); and Pediatrics, 6 (4.8%).

**Figure 1:**
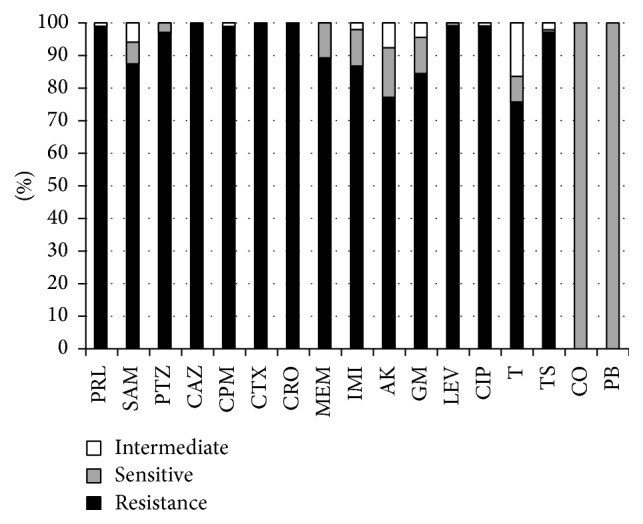
Antimicrobial susceptibility test of 124* A. baumannii* isolates in Iran. PRL: piperacillin; SAM: ampicillin-sulbactam; PTZ: piperacillin-tazobactam; CAZ: ceftazidime; CPM: cefepime; CTX: cefotaxime; CRO: ceftriaxone; MEM: meropenem; IMI: imipenem; AK: amikacin; GM: gentamicin; LEV: levofloxacin; CIP: ciprofloxacin; T: tetracycline; TS: trimethoprim-sulfamethoxazole; CO: colistin; PB: polymyxin B.

**Figure 2:**
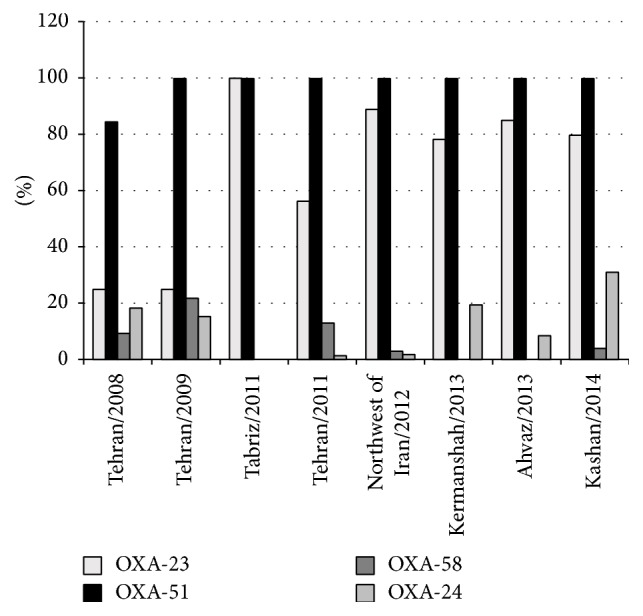
Frequency rates of *bla*_OXA_ genes in* A. baumannii* isolates in Iran (2008–2014).

**Figure 3:**
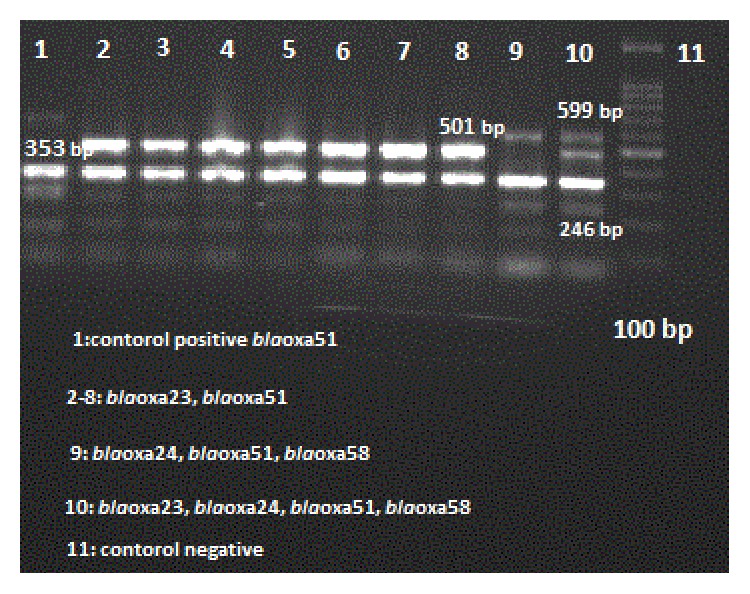
Amplification for presence of oxacillinases genes by multiplex PCR in* A. baumannii* isolates in Iran.

*Antimicrobial Resistance Pattern*. All strains were resistant to ceftazidime, ceftriaxone, and cefotaxime, while they were susceptible to colistin and polymyxin B. The resistance patterns of all* A. baumannii* isolates are shown in Figure 1. All of the* A. baumannii* isolates were considered as multidrug resistance (MDR), and 81 (96%) isolates were extensively drug resistant (XDR). Fortunately, none of the isolates were pan-drug resistant (PDR). Seven (5.6%) of isolates were ESBL positive and 68 (54.8%) isolates produced metallo-*β*-lactamases (MBLs). In our study, all of the* A. baumannii* isolates were positive for *bla*_OXA-51_ and ISA*ba*1. Analysis of incidence for OXA encoding genes of isolates demonstrated that 79.8% of them were positive for *bla*_OXA-23_, 25% for *bla*_OXA-24_, and 3.2% for *bla*_OXA-58_. The coexistence of different *bla*_OXA_ genes in isolates was observed, and their relative amounts were as follows: *bla*_OXA-51_ + *bla*_OXA-23_ in 75 (60.5%); *bla*_OXA-51_ + *bla*_OXA-24_ in 7 (5.6%); *bla*_OXA-51_ + *bla*_OXA-58_ in 3 (2.4%); *bla*_OXA-51_ + *bla*_OXA-23_ + *bla*_OXA-24_ in 23 (18.5%), and *bla*_OXA-51_ + *bla*_OXA-23_ + *bla*_OXA-24_ + *bla*_OXA-58_ in 1 isolate (0.8%). Amplification for the presence of oxacillinases genes is shown in Figure 2. *bla*_PER-1_ of Ambler class A*β*-lactamase-encoding gene was identified in 65 (52.4%) of the isolates.  Table 2 presents genetic and phenotypic analyses of isolates.  Figure 3 shows the variance in the size of amplicon products of the multiplex PCR assay for different OXA genes.

## 4. Discussion

The results of this study showed that the 124* A. baumannii* isolates were extremely resistant to most common antibiotics, surprisingly, 100% to ceftazidime, ceftriaxone, and cefotaxime. In addition, 100% of isolates were MDR, 96% XDR, and 54.8% MBL producers. In comparison, the frequency rate of ESBL producing* A. baumannii* (5.6%) was not notable in the present study. According to the previous reports, high resistance rates to carbapenems have been reported in Iran, ranging from 25% to 96.1% [10–15]. Carbapenems have been the drugs of choice for the treatment of nosocomial infections caused by* A. baumannii*; however carbapenem-resistant strains of* A. baumannii* have been reported worldwide [16]. The extensive prescription of carbapenems in our hospitals in order to treat* A. baumannii* infections has led to occurrences of carbapenem-resistant isolates. Extreme use of third-generation cephalosporins and aztreonam has exacerbated the problem of carbapenem resistance rate. In hospitals with high rates of resistance to the broad-spectrum cephalosporin, a combination of beta-lactam/beta-lactamase inhibitors, or a carbapenem, selection of appropriate antibiotics is critical. Production of carbapenem-hydrolyzing*β*-lactamases which is distributed worldwide is the major mechanism of resistance to carbapenems [17]. Polymyxin B and colistin are the most commonly used agents for* Acinetobacter* isolates which are resistant to first-line agents. The results of this study showed that 100% of the isolates were sensitive to polymyxins. It was found that 100% of* A. baumannii* were sensitive to colistin in Algeria, 70.9% in Saudi Arabia, 92.5% in Kuwait, and 95% in Egypt [18]. Epidemics of isolates harboring genes encoding OXA-type carbapenemase (*bla*_OXA-23_, *bla*_OXA-24_, *bla*_OXA-51_, and *bla*_OXA-58_ groups) have increasingly been described worldwide [19]. In the present study, 79.8% of* A. baumannii* isolates were positive for *bla*_OXA-23_ gene and more than 80% of them were resistant to both imipenem and meropenem. There are also studies on susceptibility test to carbapenems, in which an isolate is susceptible to meropenem but resistant to imipenem and vice versa; some studies have recently reported that *bla*_OXA-23_ is the most frequent type of carbapenemase identified among carbapenem-resistant* A. baumannii* [20]. The prevalence rate of *bla*_OXA-23_ has been detected from 0 to 98.4%; *bla*_OXA-51_ and ISA*ba*1 were observed in all isolates. The prevalence rate of *bla*_OXA-24/40_ has been detected from 0 to 85.43%, while the rate for *bla*_OXA-58_ has been reported from 0% to 96.9% in Colombia, Greece, Korea, Poland, Taiwan, and Turkey [13, 18]. The comparative frequency rates for *bla*_OXA_ genes in* A. baumannii* isolates in Iran are shown in Figure 2 [7, 12, 13, 21–23]. *bla*_PER-1_ was detected in 52.4% of the* A. baumannii* isolates. It was also found in* Acinetobacter* isolates from Argentina, Belgium, Egypt, France, India, Iran, Saudi Arabia, and South Korea [18, 24]. In addition, 48 isolates (48.5%) simultaneously were positive for both *bla*_OXA-23_ and *bla*_PER-1_ genes. Empiric antibiotic therapy for* Acinetobacter* isolates should be selected based on local susceptibility patterns, which consists of a broad-spectrum cephalosporin, a combination of beta-lactam/beta-lactamase inhibitors, or a carbapenem. In infectious cases with occurrence of highly antibacterial resistance rates, it is recommended to use a combination of the above agents with an antipseudomonal fluoroquinolone, an aminoglycoside, or colistin to impede the chance of treatment failure. With consideration of resistance rates to the first-line antibacterial agents, therapeutic choices are mandatory limited to polymyxins, minocycline, and tigecycline.

## 5. Conclusions

The results of the present study indicated the occurrence of high prevalence of MDR* A. baumannii* and signifies the alarming spreads of *bla*_OXA-23_ carbapenemase in our setting. On the whole, these findings recommend that screening can provide helpful information for comparisons of MDR genetic collection between different populations and countries and potentially support more infection control strategies and chemotherapeutic treatment programs.

## Figures and Tables

**Table 1:** Primers used in this study for amplification of genes from *Acinetobacter baumannii* isolates.

Primer	Nucleotide sequence (5′ to 3′)	Amplicon size (bp)	Reference
OXA51LF	TAA TGC TTT GAT CGG CCT TG	353	Feizabadi et al. (2008) [7]
OXA51LR	TGG ATT GCA CTT CAT CTT GG		

OXA23LF	GAT CGG ATT GGA GAA CCA GA	501	Feizabadi et al. (2008) [7]
OXA23LR	ATT TCT GAC CGC ATT TCC AT		

OXA24LF	GGT TAG TTG GCC CCC TTA AA	246	Feizabadi et al. (2008) [7]
OXA24LR	AGT TGA GCG AAA AGG GGA TT		

OXA58LF	AAGTATTGGGGCTTGTGCTG	599	Feizabadi et al. (2008) [7]
OXA58LR	CCCCTCTGCGCTCTACATAC		

ISAba1F	CACGAATGCAGAAGTTG	549	Segal et al. (2005) [8]
ISAba1R	CGACGAATACTATGACAC		

PER-1 F	ATGAATGTCATTATAAAAGC	925	Vahaboglu et al. (2001) [9]
PER-1 R	AATTTGGGCTTAGGGCAGAA		

**Table 2:** Summary of resistance: phenotypic and genetic characteristics of the isolates^a^.

Pattern	Resistance phenotype^b^																		*bla *genes					
	No	PRL	SAM	PTZ	CAZ	CPM	CTX	CRO	MEM	IMI	AK	GM	LEV	CIP	T	TS	CO	PB	OXA-23	OXA-24	OXA-51	OXA-58	PER-1	ISAab1
1	25	+	+	+	+	+	+	+	+	+	+	+	+	+	+	+	−	−	+	−	+	−	−	+
2	16	+	+	+	+	+	+	+	+	+	+	+	+	+	+	+	−	−	+	−	+	−	+	+
3	10	+	+	+	+	+	+	+	+	+	+	+	+	+	+	+	−	−	+	+	+	−	+	+
4	9	+	+	+	+	+	+	+	+	+	+	+	+	+	+	+	−	−	+	+	+	−	−	+
5	8	+	+	+	+	+	+	+	+	+	+	+	+	+	+	+	−	−	+	+	+	−	−	+
6	5	+	+	+	+	+	+	+	+	+	−	+	+	+	+	+	−	−	+	−	+	−	+	+
7	4	+	−	+	+	+	+	+	−	−	+	+	+	+	+	+	−	−	−	−	+	−	+	+
8	3	+	+	+	+	+	+	+	+	+	−	−	+	+	+	+	−	−	+	−	+	−	−	+
9	3	+	+	+	+	+	+	+	+	+	+	+	+	+	−	+	−	−	+	−	+	−	−	+
10	2	+	+	+	+	+	+	+	+	+	−	+	+	+	+	+	−	−	+	−	+	−	−	+
11	2	+	−	+	+	+	+	+	+	+	+	+	+	+	−	+	−	−	+	−	+	−	−	+
12	2	+	+	+	+	+	+	+	+	+	+	+	+	+	−	+	−	−	−	−	+	−	+	+
13	2	+	+	+	+	+	+	+	+	+	+	+	+	+	+	+	−	−	−	+	+	−	−	+
14	1	+	+	+	+	+	+	+	+	+	+	−	+	+	−	+	−	−	+	+	+	−	+	+
15	1	+	+	+	+	+	+	+	+	+	+	−	+	+	+	+	−	−	+	+	+	−	+	+
16	1	+	+	+	+	+	+	+	+	+	+	+	+	+	+	+		−	−	+	+	−	+	+
17	1	+	−	+	+	+	+	+	+	+	−	+	+	+	+	+	−	−	−	+	+	−	+	+
18	1	+	+	+	+	+	+	+	+	+	−	+	+	+	−	+	−	−	−	+	+	−	+	+
19	1	+	+	+	+	+	+	+	+	+	−	+	+	+	+	+	−	−	−	+	+	−	+	+
20	1	+	+	+	+	+	+	+	+	+	+	+	−	−	−	+	−	−	−	+	+	−	−	+
21	1	+	+	−	+	−	+	+	−	−	−	−	+	+	−	−	−	−	−	−	+	+	+	+
22	1	+	+	+	+	+	+	+	+	−	+	+	+	+	+	+	−	−	−	−	+	+	+	+
23	1	+	+	+	+	+	+	+	+	+	+	+	+	+	−	+	−	−	−	−	+	+	−	+
24	1	+	+	+	+	+	+	+	+	+	−	−	+	+	+	+	−	−	+	+	+	−	−	+
25	1	+	−	+	+	+	+	+	+	+	−	+	+	+	+	+	−	−	+	+	+	−	−	+
26	1	+	−	+	+	+	+	+	+	+	−	−	+	+	+	+	−	−	+	+	+	−	−	+
27	1	+	+	+	+	+	+	+	+	+	+	+	+	+	+	+	−	−	+	+	+	+	−	+
28	1	+	−	+	+	+	+	+	−	−	−	−	+	+	−	−	−	−	−	−	+	−	−	+
29	1	+	+	+	+	+	+	+	+	−	−	+	+	+	+	+	−	−	−	−	+	−	−	+
30	1	+	−	−	+	+	+	+	−	−	−	−	+	+	−	−	−	−	−	−	+	−	−	+
31	1	+	−	+	+	+	+	+	−	−	+	−	+	+	−	+	−	−	−	−	+	−	−	+
32	1	+	+	+	+	+	+	+	−	−	+	+	+	+	+	+	−	−	−	−	+	−	+	+
33	1	−	+	+	+	+	+	+	−	−	−	+	+	+	+	+	−	−	−	−	+	−	+	+
34	1	+	+	+	+	+	+	+	+	−	−	+	+	+	+	+	−	−	−	−	+	−	+	+
35	1	+	−	−	+	+	+	+	−	−	−	−	+	+	+	+	−	−	−	−	+	−	+	+
36	1	+	+	+	+	+	+	+	−	+	+	+	+	+	−	+	−	−	−	−	+	−	+	+
37	1	+	+	+	+	+	+	+	+	+	−	+	+	+	−	+	−	−	+	−	+	−	+	+
38	1	+	+	+	+	+	+	+	+	+	−	−	+	+	+	+	−	−	+	−	+	−	+	+
39	1	+	+	+	+	+	+	+	+	+	+	−	+	+	+	+	−	−	+	−	+	−	+	+
40	1	+	−	+	+	+	+	+	−	+	+	−	+	+	−	+	−	−	+	−	+	−	+	+
41	1	+	+	+	+	+	+	+	+	−	−	+	+	+	−	+	−	−	+	−	+	−	+	+
42	1	+	+	+	+	+	+	+	+	+	+	−	+	+	−	+	−	−	+	−	+	−	−	+
43	1	+	+	+	+	+	+	+	+	+	+	−	+	+	+	+	−	−	+	−	+	−	−	+
44	1	+	+	+	+	+	+	+	+	−	+	+	+	+	+	+	−	−	+	−	+	−	−	+
45	1	+	+	+	+	+	+	+	+	+	−	−	+	+	−	+	−	−	+	−	+	−	−	+
46	1	+	−	+	+	+	+	+	+	−	−	+	+	+	+	+	−	−	+	−	+	−	−	+

^a+^Presence of indicated gene or resistance phenotype.

^b^PRL: piperacillin; SAM: ampicillin-sulbactam; PTZ: piperacillin-tazobactam; CAZ: ceftazidime; CPM: cefepime; CTX: cefotaxime; CRO: ceftriaxone; MEM: meropenem; IMI: imipenem; AK: amikacin; GM: gentamicin; LEV: levofloxacin; CIP: ciprofloxacin; T: tetracycline, TS: trimethoprim-sulfamethoxazole; CO: colistin; PB: polymyxin B.
